# Professional Identity and Motivation for Medical School in First-Year Medical Students: A Cross-sectional Study

**DOI:** 10.1007/s40670-023-01754-7

**Published:** 2023-03-06

**Authors:** Valentina Faihs, Susanne Heininger, Stuart McLennan, Martin Gartmeier, Pascal O. Berberat, Marjo Wijnen-Meijer

**Affiliations:** 1 TUM Medical Education Center, TUM School of Medicine, Technical University of Munich, Ismaninger Str. 22, 81675 Munich, Germany; 2 Department of Dermatology and Allergy Biederstein, TUM School of Medicine, Technical University of Munich, Biedersteiner Str. 29, 80802 Munich, Germany; 3 Institute of History and Ethics in Medicine, TUM School of Medicine, Technical University of Munich, Ismaninger Str. 22, 81675 Munich, Germany

**Keywords:** Professional identity, Motivation, Medical education, Medical students

## Abstract

**Background:**

Professional identity formation (PIF) is a life-long process, starting even before professional education. High levels of motivation for medical school are essential for effective learning and academic success. Both are key factors in future physicians’ professional and personal development, and according to self-determination theory, professional identity (PI) and students’ levels of motivation could be closely linked. Therefore, we sought to investigate whether PI and strength of motivation for medical school are associated in new medical students.

**Methods:**

In a cross-sectional survey, all new medical students in Munich, Germany, were asked to complete the Macleod Clark Professional Identity Scale (MCPIS-9) and the Strength of Motivation for Medical School-Revised questionnaire (SMMS-R) as well as to provide information about age, gender, and waiting time before starting medical school.

**Results:**

Eight hundred eleven out of 918 new medical students participated in the survey. A positive correlation between the MCPIS-9 and the SMMS-R (*p* < 0.001) was found. Female students showed higher scores in the SMMS-R (*p* < 0.05) and the SMMS-R-subscale *Readiness to Start* (*p* < 0.001)*.* The amount of waiting semesters showed a positive correlation with the total SMMS-R score (*p* < 0.01) as well as with the subscales *Readiness to Start* and *Persistence* (both *p* < 0.001).

**Discussion:**

We found an association between PI and strength of motivation for medical school in a large cohort of new medical students. Female gender and more waiting semesters were associated with higher levels of self-perceived motivation and higher scores on the SMMS-R-subscale *Readiness to Start*. More research is needed to better understand this topic to further improve medical education.

## Introduction

### Professional Identity (PI) and Professional Identity Formation (PIF)

In recent years, professional identity formation (PIF) in medical students has received increasing attention [1–8]. Professional identity (PI) can be described as “the attitudes, values, knowledge, beliefs and skills that are shared with others within a professional group” [9]. According to Adams and colleagues, PI—seen as a measure of group identification—is evident in students even before they begin their professional training and undergoes a process of continuous development (professional identity formation, PIF) [9].

PIF is a continuous process, beginning way before medical university and going on throughout the whole career [10]. This process happens simultaneously on an individual (psychological) and on a collective level, meaning a person’s socialization into appropriate roles and professional relationships [11, 12]. On the collective level, especially, interactions with members of the medical profession who are seen as role models or mentors have a significant impact [13, 14]. On the individual level, PIF is shaped by various factors like personal background, relationships, and expectations [15]. Importantly, literature also indicates a link between students’ autonomous motivation and a positive PI [16, 17].

An emerging body of literature claims that enhancing PIF should be a principal goal of medical education alongside acquiring clinical skills and knowledge [18–21]. Jarvis-Selinger and colleagues suggest moving the focus of medical education from “*doing* the work of a physician” towards “*being* a physician” [10]. Accordingly, Cruess et al. proposed an amendment to the Miller’s pyramid, illustrating a structured approach for the assessment of medical competence: in addition to the original structure including the layers “Knows,” “Knows How,” “Shows How,” and “Does” from the bottom to the top of the pyramid, a fifth level with “Is” indicating PI is proposed as the highest level of accomplishment at the apex of the pyramid [22, 23]. However, we argue that PI does not emerge when all different aspects of medical professionalism (knowledge, practical skills, attitudes) have been successfully developed; instead, it is a continuous development and formation process [24].

On the one hand, PIF is of great importance for medical students’ subjective well-being [25]. Studies have shown that the identity of being a doctor is more important to medical students’ sense of self than their identity as students [26]. Among nursing students, a high PI was closely linked with student retention in the education program [27]. On the other hand, PIF is essential for future physicians’ professional development as it helps prevent role confusion, particularly when working with colleagues of many different professions [10, 18–23, 25–28]. This aspect plays a vital role in the context of interprofessional education [9]. Also, a strong PI is essential for physicians’ good relationship with their patients, helping them practice confidently and professionally [18, 20, 29].

### Approaches to Enhance PIF in Medical School

To support PIF in medical school, different measures have been proposed. It has been shown that guided reflection, in a group as well as individually, can be a precious tool to enhance PIF in medical students [7, 12, 30–32]. Additionally, teaching modules focusing on medical ethics and the possibility of creative and emotional expression (i.e., through arts or stories) may support medical students’ PIF [30, 31]. Of course, early practical experiences and early contact with role models can also strengthen medical students’ PIF [32]. Recently, Mount and colleagues showed that reflective writing seems to be the most commonly used tool to support PIF in medical school [33]. However, the authors emphasize that this measure mainly focuses on an individualist perspective on PIF, while PIF happens on a social-contextual level, too [33]. Other measures able to strengthen PIF in medical students with a stronger focus on the social context of PIF are mentoring programs [34, 35] or the establishment of interprofessional learning sessions [12, 36].

### Self-Determination Theory (SDT), PIF, and Motivation

Mylrea and colleagues proposed self-determination theory (SDT) to approach PIF and its link to students’ motivation [17]. SDT, developed by psychologists Ryan and Deci, is among the major psychological theories explaining motivational processes and can provide many implications for medical education [37–40]. SDT describes a continuum of motivation types with their regulators and causality bases [41]. Human motivation is described as a continuum ranging from absent motivation (amotivation) to extrinsic motivation to the most autonomous type of intrinsic motivation, leading an individual to engage purely by internal forces like interest or enjoyment [17, 41]. Based on this construct, a relationship between high (intrinsic) motivation for an activity and the incorporation into a person’s sense of self (individual internalization) is stated, supporting the theory that increased levels of motivation lead to an enhanced PIF [17, 42]. This means that an intrinsically motivated person will act highly autonomously because the activity has become part of their individual identity, PI in the case of medical education [17, 42, 43]. On the contrary, PIF can in turn enhance students’ motivation for their studies [44, 45]. However, to date, little is known about the associations between PI, PIF, and strength of motivation in medical students.

### Motivation for Medical School

Accordingly, Kusurkar and colleagues state that motivation for medical studies can be both a dependent and an independent variable [34]. As a dependent variable, motivation could be influenced or enhanced by factors like changes in the curriculum and the learning environment [46] or, as explained before, by PI. As an independent variable, it is known that the motivation of students stimulates learning and enhances their academic success [46–49]. Moreover, high levels of intrinsic motivation are associated with greater subjective well-being and meaning in life of students [50, 51]. Thus, a better understanding of factors influencing students’ motivation is of great importance for medical education [34, 46].

Although numerous sociocultural factors seem to influence strength of motivation for medical school, more research is needed to better understand such relationships [52]. Accordingly, studies on gender differences in students’ motivation are rather contradictory [52]. Similarly, more research is needed on how factors like gender and prior education affect PIF on an individual level [53].

### The Waiting Time Quota for Medical School Admission in Germany

In Germany, admission to medical school is made by different quotas. In 2019, 20% of medical students were admitted by waiting time or “waiting semesters” (equivalent to half a year), 20% by excellent school-leaving grades, and 60% through the university’s selection process, which in Bavaria considers school-leaving grades as well as a completed vocational training (sometimes resulting in a low number of “waiting semesters” even in this quote) or an entry test. For admission through the waiting time quota, those who have waited for the longest since high school graduation without enrolling in a public university have the greatest chances of getting admitted [54]. While no academic study at a public university in Germany is allowed during the waiting time, a vocational training can be pursued. In 2019, 14 waiting semesters, equivalating to 7 years, were needed to be admitted through this quota [55]; some students, however, bring a far higher number of waiting semesters, for instance, if they actively decided to keep working in another sector for several years before entering medical school. This system leads to an increased number of students with previous professional experiences, as many decide to pursue vocational training in the meantime [49]. Especially, students with a prior career in the medical sector—for example in nursing or physiotherapy—start their medical education with valuable experiences in this field; however, studies suggest that these students may have difficulties in negotiating their new identity as (future) doctors instead of their previously gained PI [56]. Studies show that graduation rates are lower in students admitted by waiting time [57]. Among other factors, the feeling of being under-challenged in their prior (medicine-related) occupation as crucial factor for motivation for taking up medical studies has been shown to be associated with higher graduation rates in these students [49].

### Study Aims

In the present study, we seek to enhance the existing literature by investigating the association between PI and the strength of motivation for medical school in students’ right at the beginning of their medical education. Thus, we sought to investigate the following questions:

- Can an association between PI and strength of motivation for medical school be found in new medical students?
- Are the factors age, gender, and waiting time before starting medical school associated with PI and/or strength of motivation for medical school?

The answer to these questions could help to a better understanding of this relevant topic which is the basis for a better implementation of methods to enhance PIF and strength of motivation for medical school in the curriculum and thus improve medical education in the future.

## Methods

### Study Context and Study Design

This study was part of a larger cross-sectional survey conducted on 14 October 2019. All new medical students at the Ludwig Maximilian University and the Technical University of Munich must complete a joint compulsory course “Introduction to Clinical Medicine” in the 2.5 days of their first semester. In the context of this course (with 918 students registered for 2019), students were invited to participate in the study; it was stressed that their participation in the study was voluntary and completely independent of the successful course completion. The paper-based survey was prepared using the software EvaSys (EvaSys Central Evaluation version 8.0). All participants gave written consent to this anonymous survey. Students were asked to complete the Macleod Clark Professional Identity Scale (MCPIS-9) [9] and the Strength of Motivation for Medical School–Revised questionnaire (SMMS-R) [34] as well as to provide information about gender, the number of waiting semesters, and the year of birth to calculate age. Approval was obtained from the Ethics Commission of the Technical University of Munich.

### The Macleod Clark Professional Identity Scale (MCPIS-9)

The MCPIS-9 is a validated instrument developed for the measurement of the PI of health and social care students from different professions, including medical students [9, 27, 58]. This scale was first developed by Adams and colleagues for the measurement of PI in first-year students before the beginning of their training [9]. The single-construct questionnaire consists of 9 items—with 3 items being reverse-scored—measured on a 5-point Likert scale. For the final score calculation, each item’s scores were added together. An overview of the original, English version of the questionnaire [9] is given in Table 1. The questionnaire was translated into German by scientists from the TUM Medical Education Center. To ensure a high-quality translation, back translation was performed.

**Table 1:** Overview on MCPIS-9 questionnaire in English

1	I feel like I am a member of this profession.	
2	I feel I have strong ties with members of this profession.	
3	I am often ashamed to admit that I am studying for this profession.	Reverse-scored
4	I find myself making excuses for belonging to this profession.	Reverse-scored
5	I try to hide that I am studying to be part of this profession.	Reverse-scored
6	I am pleased to belong to this profession	
7	I can identify positively with members of this profession.	
8	Being a member of this profession is important to me	
9	I feel I share characteristics with other members of the profession.	

### The Strength of Motivation for Medical School–Revised Questionnaire (SMMS-R)

The SMMS-R is a validated instrument specially developed for the measurement of the strength of motivation for medical studies [34, 59, 60]. Of note, it does not measure the type of motivation. This scale can be a valuable tool to measure the outcome of educational interventions to improve motivation, to describe the characteristics of a medical students’ cohort, or to assess the quality of a selection process [34, 59, 60]. Measurement invariance has been demonstrated, so it can be used for group and cross-cultural comparisons [61]. The SMMS-R consists of 15 items on 5-point Likert scales and has been shown to have a three-factor structure from statistical as well as theoretical viewpoints with following subscales: *Willingness to Sacrifice, Readiness to Start*, and *Persistence* [60, 62]. Seven negatively formulated items have to be reverse-scored. The scores of each item are added for calculation of the subscales and the SMMS-R total score, with higher scores indicating a greater strength of motivation. An overview of the English version of the questionnaire [34] is given in Table 2. The SMMS-R was translated in German in a multi-step translation process (translation of the Dutch and English versions of the questionnaire in German by separate scientists of TUM Medical Education Center, followed by comparison of both German versions by a third scientist).

**Table 2:** Overview on SMMS-R questionnaire in English

**No.**	**SMMS-R items**	
***Subscale: Willingness to Sacrifice***		
5	Even if I could hardly maintain my social life, I would still continue medical training.	
7	I would still choose medicine even if that meant I would never be able to go on holidays with my friends anymore.	
9	If studying took me more than an average of 60 h a week**,** I would seriously consider quitting.	Reverse-scored
10	I intend to become a doctor even though that would mean taking CME courses two evenings a week throughout my professional career.	
***Subscale: Readiness to Start***		
1	I would always regret my decision if I hadn’t availed myself of the opportunity to study medicine.	
3	I would still choose medicine even if that would mean studying in a foreign country in a language that I have not yet mastered.	
6	I wouldn’t consider any other profession than becoming a doctor.	
11	It wouldn’t really bother me too much if I could no longer study medicine.	Reverse-scored
15	I would be prepared to retake my final high school exams to get higher marks if this would be necessary to study medicine.	
***Subscale: Persistence***		
2	I would quit studying medicine if I were 95% certain that I could never become the specialist of my choice.	Reverse-scored
4	As soon as I would discover that it would take me ten years to qualify as a doctor, I would stop studying.	Reverse-scored
8	I would stop studying medicine if I started scoring low marks and failing tests often.	Reverse-scored
13	I would quit studying as soon as it became apparent that there were no jobs or resident positions after graduation.	Reverse-scored
14	I would not have chosen medicine if it would have caused me to accumulate substantial financial debts.	Reverse-scored

### Data Analysis

Statistical analysis was performed with IBM SPSS Statistics 27 software (SPSS Incorporated, Chicago, USA). Descriptive statistics were calculated for demographic data, MCPIS-9 and SMMS-R. MCPIS-9 and SMMS-R were obtained only for individuals who completed all items of the scales, respectively, each subscale of the SMMS-R. Cronbach’s alpha was calculated for both scales and the three-factor structure of SMMS-R was checked by principal components analysis using Promax rotation with Kaiser normalization. Kolmogorov–Smirnov test and Shapiro–Wilk test were performed to test for normal distribution. Non-normally distributed data are given as median and interquartile range (IQR). Spearman’s rank correlation coefficient was used to calculate bivariate correlations. Mann–Whitney *U* test was used for differences between groups. A *p*-value of < 0.05 was considered statistically significant.

## Results

A total of 811 out of 918 new medical students participated in the survey (response rate 88.3%). Sixty-seven percent of the students identified themselves as female (*n* = 492), 32.8% as male (*n* = 241), and 0.1% (*n* = 1) as non-binary (*n* = 182 missing data). The median age was 20.0 years (IQR 3.0 years, *n* = 139 missing values). The median waiting time was 2.0 semesters (IQR 4.0 semesters; mean 4.94 semesters, standard deviation 10.3 semesters; no significant gender difference). As expected, the amount of waiting semesters correlated with the students’ age (Spearman’s rho = 0.633, *p* < 0.001).

Cronbach’s alpha was acceptable with 0.795 for MCPIS-9 and 0.785 for SMMS-R. In the factor analysis with Promax rotation, the three separate SMMS-R-subscales could be verified. The three subscales explained 43.0% of the variance of SMMS-R-scores (*Willingness to Sacrifice:* Eigenvalue 3.99, explained 26.59% of the variance of SMMS-R; *Readiness to Start*: Eigenvalue 1.27, explained 8.45% of the variance; *Persistence:* Eigenvalue 1.19, explained 7.96% of the variance).

As shown in Table 3, the median total score for the MCPIS-9 was 39.0 (IQR 6.0, mean value 38.7) and the median total score for the SMMS-R was 56.0 (IQR 11.2, mean 56.2).

**Table 3:** Overview on MCPIS-9 and SMMS-R with subscales

		**Median**	**IQR**	***p*-value**	***n***
**MCPIS-9**	Total	39	6		660
	Female	39	6	0.270	401
	Male	40	6		189
**SMMS-R**	Total	56	11		506
	Female	57	11	**0.011**	322
	Male	54	12		162
**Subscale Willingness to Sacrifice**	Total	19	5		570
	Female	19	5	0.274	363
	Male	19	7		173
**Subscale Readiness to Start**	Total	19	5		584
	Female	19	6	** < 0.001**	373
	Male	18	6		179
**Subscale Persistence**	Total	19	4		569
	Female	19	4	0.220	358
	Male	19	5		181

Mann–Whitney *U* test for gender differences. Total respondents’ numbers include data of students with missing data on gender

*MCPIS-9* Macleod Clark Professional Identity Scale, *SMMS-R* Strength of Motivation for Medical School–Revised questionnaire, *IQR* interquartile range

**Fig. 1:**
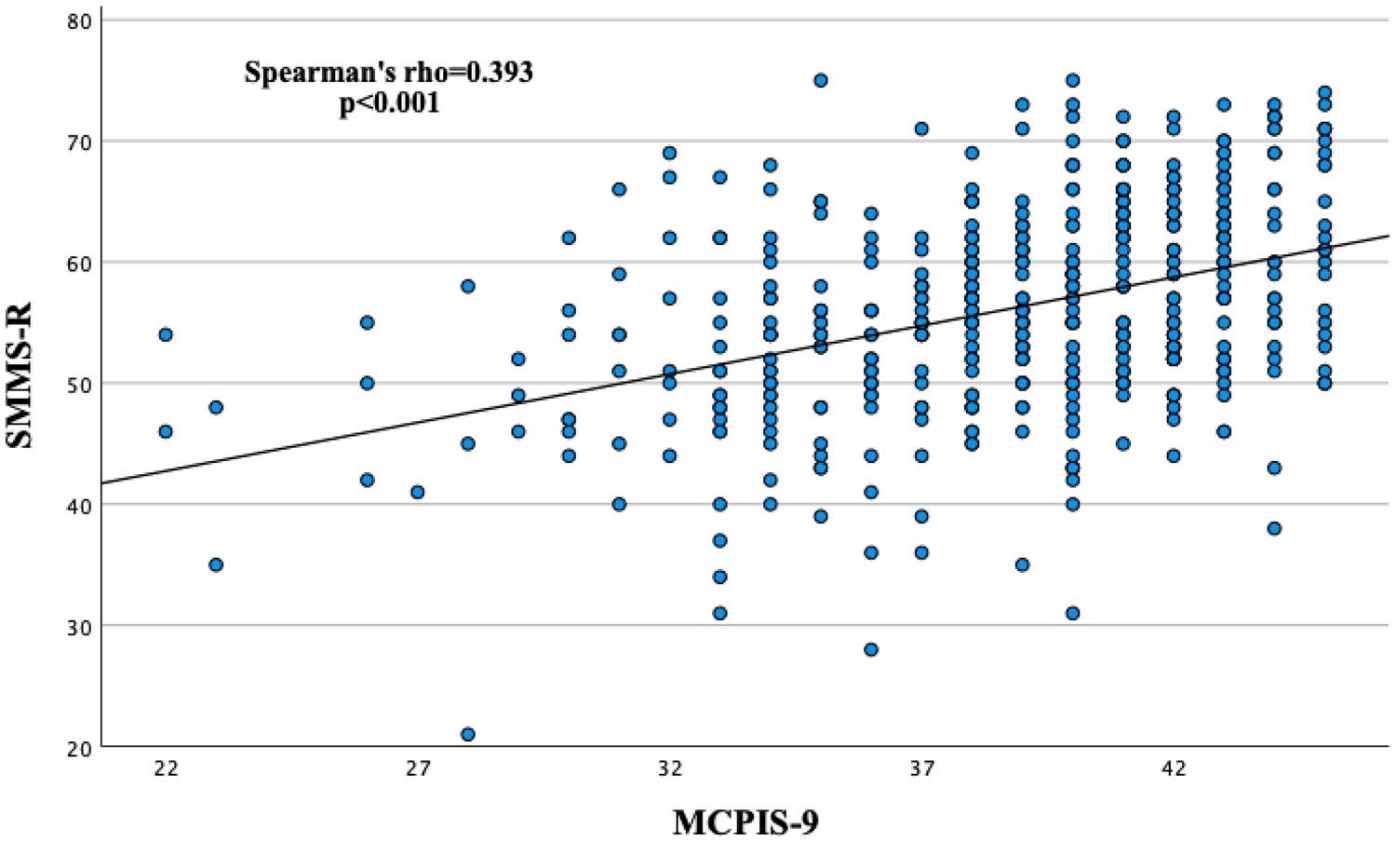
Correlation between the Macleod Clark Professional Identity Scale (MCPIS-9) and the Strength of Motivation for Medical School–Revised questionnaire (SMMS-R) in first-year medical students

**Fig. 2:**
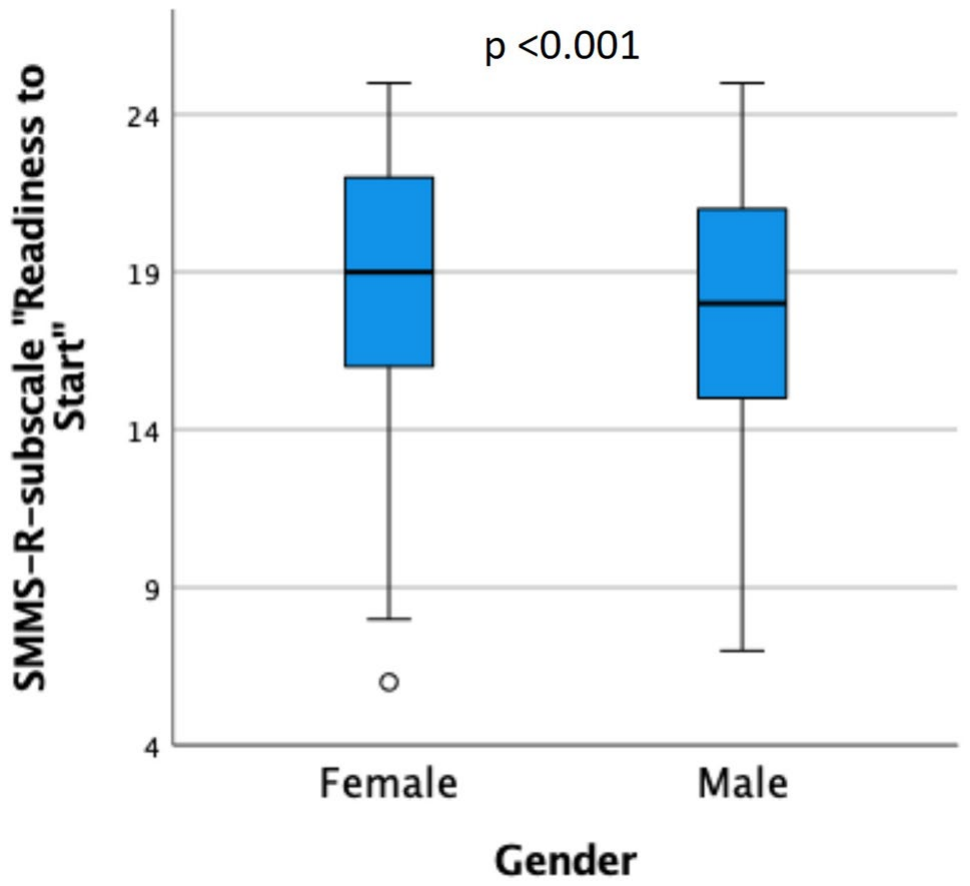
Scores in the Strength of Motivation for Medical School–Revised questionnaire (SMMS-R)-subscale “*Readiness to Start*” of female and male first-year medial students. Difference analyzed with Mann–Whitney *U* test

We found a moderate, positive correlation between the MCPIS-9 and the SMMS-R (Spearman’s rho = 0.393, *p* < 0.001; Fig. 1). For MCPIS-9, no significant associations with gender, age, or the amount of waiting semesters were found. However, female students showed higher total scores in the SMMS-R than male students (*p* < 0.05, Table 3). Female students showed significantly higher scores in the subscale *Readiness to Start* (*p* < 0.001, Table 3 and Fig. 2), while no gender difference was found for the other subscales.

**Fig. 3:**
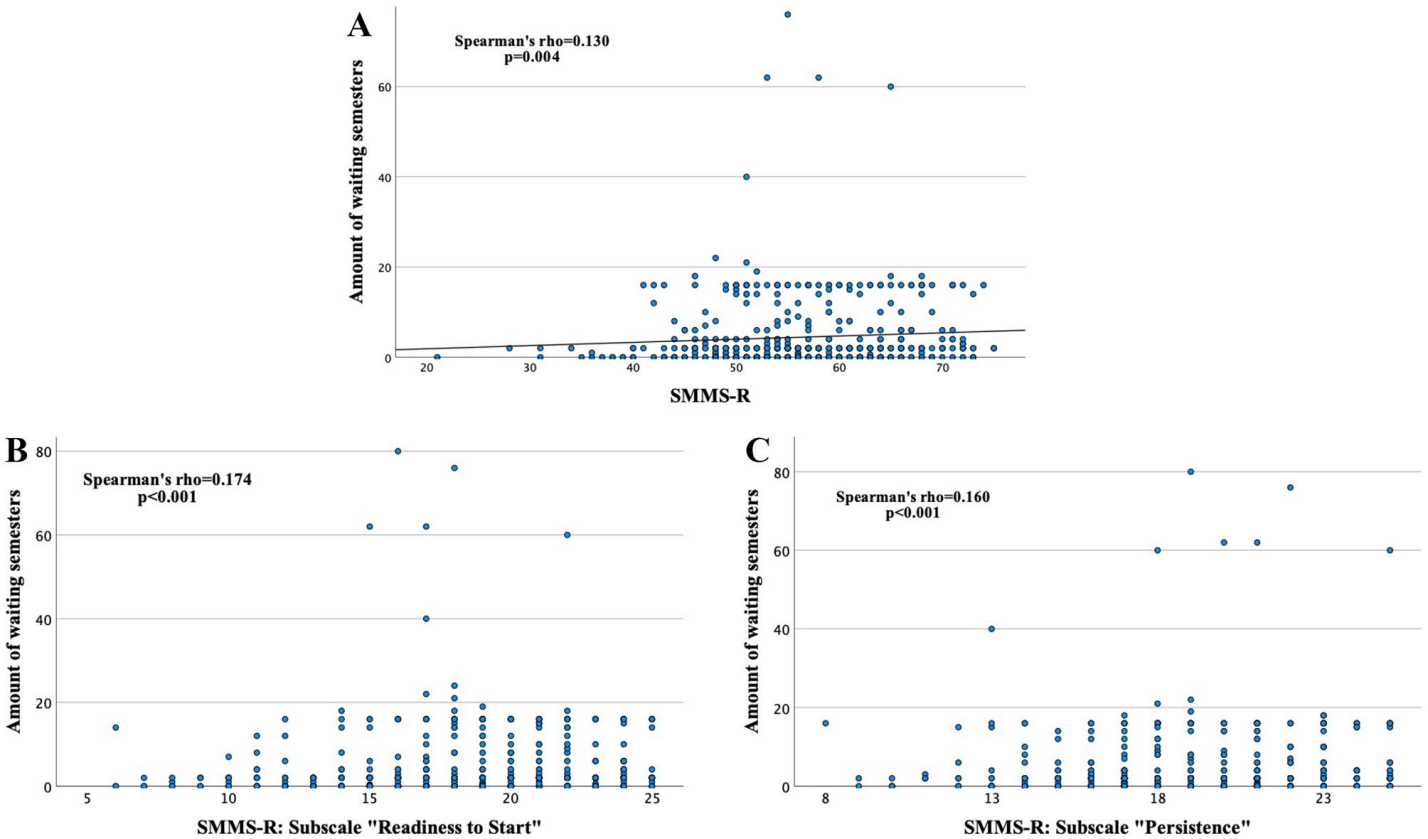
Correlation between the amount of waiting semesters and the SMMS-R total score (**A**) as well as with the SMMS-R-subscales *Readiness to Start* (**B**) and *Persistence* (**C**) in first-year medical students

The amount of waiting semesters showed a positive correlation with the total SMMS-R score (Spearman’s rho = 0.130, *p* = 0.004; Fig. 3A) as well as with the two SMMS-R-subscales *Readiness to Start* (Spearman’s rho = 0.174, *p* < 0.001; Fig. 3B) and *Persistence* (Spearman’s rho = 0.160, *p* < 0.001; Fig. 3C). For age, a weak correlation was found with the SMMS-R-subscale *Readiness to Start* (Spearman’s rho = 0.094, *p* = 0.035), whereas there was no further correlation with SMMS-R total score or subscales, or MCPIS-9.

## Discussion

This was the first study analyzing whether motivation for medical school shows an association to PI in students entering medical school. This study found a moderate, positive correlation between PI and strength of motivation for medical school in new medical students measured with MCPIS-9 and SMMS-R, respectively. This is in line with the hypothesis that PI and motivation for medical studies are closely linked, as is supported by the theoretical principles of SDT. As described above, SDT can be used to describe the internalization process leading to PIF when high motivation is displayed for certain activities, like medical studies [17, 42]. So, our results align with SDT, as higher levels of motivation were positively associated with higher levels of PI in first-year medical students. However, it is also plausible that a good PI can enhance students’ motivation [44, 45]. Our data provide additional evidence for the close relationship between levels of PI and motivation levels in new medical students.

These results are in line with studies finding a moderate correlation between motivation levels in pharmacy students and PI measured with MCPIS-9 [17]. However, it is impossible to determine whether one of the measures is a dependent variable. As described by other studies, both the MCPIS-9 [9, 27, 58] and the SMMS-R [34, 60] showed adequate internal consistency. Moreover, we could confirm the three-factor structure of SMMS-R [34].

It should be of great interest for medical educators to enhance the strength of motivation [37, 38, 46, 50] as well as PIF [14, 20, 21, 29, 34] of students to support their academic, personal, and professional development. At the Technical University of Munich, different measures and courses supporting PIF have been established, including a program for medical humanities (LET ME) inviting to guided reflection and discussions, as well as a mentoring program and different mandatory and facultative courses offering discussion rounds and ethics teaching. Additionally, early practical and clinical experiences are facilitated by single courses with real patients at the hospital already in the preclinical study years.

Considering this study’s results showing an association between students’ motivation and PI, one could hypothesize that measures to strengthen students’ PI, for example mentoring programs [34, 35] or the establishment of interprofessional learning sessions [36], are at the same time able to strengthen students’ motivation for medical school. In the same way, measures to enhance students’ motivation, for instance making them solve problems in the learning process or strengthening the link between theory and practice in medical education [63], could potentially enhance their PIF. Further research is needed to verify these associations in longitudinal, interventional studies. All in all, this study’s results support the need for the implication of these measures in medical education, as they may both have an effect for the enhancement of PIF and students’ motivation.

The total scores of both questionnaires in our study showed comparable or even slightly higher levels of strength of motivation [42, 46, 52, 61] and PI [42, in pharmacy students] of new medical students in Munich when compared to similar surveys conducted in different study years. The time point right at the beginning of medical education may be an important factor: the strength of motivation for medical school seems to be highest at the beginning and to slightly decrease during the first year of university [26, 59]. Also, a demanding selection procedure may temporarily enhance the student’s motivation [64].

67% of respondents in the survey were female—this is in line with over 60% of admitted medical students being female in Munich over the last years. In our survey, female students showed higher scores in the total SMMS-R and in the SMMS-R-subscale *Readiness to Start*. Similar results were found among medical students in the Netherlands [52], while a recent study in China showed higher intrinsic motivation levels among male students [65]. One could hypothesize that the higher motivation rates among females may be one possible cause for the higher admission rates of females to medical school in Germany, while the underlying causes remain elusive. More research is needed to elucidate the role of gender and cultural background for strength of motivation for medical studies. A response bias with females answering more confidently in surveys seems highly unlikely, as in several questionnaire studies female medical students were shown to self-assess themselves as worse than their male peers, while in studies assessing their practical skills, no objective differences could be found [66–69]. As depicted above, literature is also contradictory regarding the role of gender in PIF [1, 9, 53]. In our study, we could not find any difference in MCPIS-9 scores between male and female students.

As already mentioned, in Germany in 2019, people without good to excellent school-leaving grades had the only possibility to enter medical school via the waiting time quota. During this waiting time, no academic study in Germany was allowed, leading many applicants to complete a vocational training in the meantime. In 2019, 14 waiting semesters were needed to be admitted through this quota [55], but some students even brought a far higher waiting time when entering medical school. However, this quota will be abolished in the future [70].

Interestingly, our data show an association between the number of waiting semesters and the strength of motivation to pursue medical studies. Looking at the SMMS-R-subscales, we found an association between the amount of waiting semesters and the readiness to start medical school, as well as the students’ self-perceived persistence for their studies. Of course, students with a higher amount of waiting semesters are older on average, but age showed only one weak correlation with SMMS-R-subscale *Readiness to Start*. The willingness to wait for many years to enter medical school can be seen as a signal for strong motivation [71]. In the already mentioned study of Kusurkar and colleagues, where waiting periods were not assessed, age was the most significant single predictor of the strength of motivation [52]. A possible explanation could be that older students may bring more certainty about their career choice [72], especially if they work in the medical sector during their waiting time. During the many years needed for the waiting list quota, less motivated students may change their career plans. However, graduation rates are lower in students admitted by waiting time—age-related factors like financial self-support or an own family may be important factors for this point [57]. Nevertheless, high motivation levels for medical studies have been shown to be associated with better study success in these students [49]. In a study at a German university, all students admitted by waiting time who had reached the clinical part of their training also reached their final year [57]. This is in line with our finding that a higher amount of waiting semesters is associated with higher scores in the SMMS-R-subscale *Persistence*. So, it seems that this association may be stable throughout the years of study and can be objectified.

While the high response rate is a strength of this study, some limitations must be mentioned. First, the study was conducted in one single city in Germany (among students from two institutions, however), limiting the generalizability of the findings. However, the conduction right at the beginning of the semester limits possible institutional influences. Second, although the SMMS-R and the MCPIS-9 are validated instruments and a multi-step translation process including back translation was performed, the German version used in this study was not re-validated. Third, no data about previous working experience or vocational training were collected. Fourth, due to this study’s cross-sectional character, we cannot provide information about the dynamics of the measured variables throughout the course of study. This would be a valuable perspective for further research in future studies.

## Conclusions

PIF and motivation are essential factors in the professional development of (future) doctors and their personal well-being. In this study, we could show that PI and strength of motivation for medical school are associated in a large cohort of new medical students. This finding is in line with the theoretical principles of SDT. Female gender and a higher amount of waiting semesters were associated with higher levels of self-perceived motivation and higher scores on the SMMS-R-subscale *Readiness to Start*, whereas no differences were found regarding PI. More research, especially longitudinal and interventional studies, are needed to better understand this topic and methods to enhance PIF and motivation in medical education.

## Data Availability

The datasets analysed during the current study are available from the corresponding author on reasonable request.

## Abbreviations

IQR: Interquartile range
MCPIS-9: Macleod Clark Professional Identity Scale
PI: Professional identity
PIF: Professional identity formation
SDT: Self-determination theory
SMMS-R: Strength of Motivation for Medical School–Revised questionnaire
